# Associations of high altitude polycythemia with polymorphisms in *EPHA2* and *AGT* in Chinese Han and Tibetan populations

**DOI:** 10.18632/oncotarget.18384

**Published:** 2017-06-06

**Authors:** Lijun Liu, Yao Zhang, Zhiying Zhang, Yiduo Zhao, Xiaowei Fan, Lifeng Ma, Yuan Zhang, Haijin He, Longli Kang

**Affiliations:** ^1^ Key Laboratory for Molecular Genetic Mechanisms and Intervention Research on High Altitude Disease of Tibet Autonomous Region, School of Medicine, Xizang Minzu University, Xianyang 712082, Shaanxi, China; ^2^ Key Laboratory of High Altitude Environment and Gene Related to Disease of Tibet Ministry of Education, School of Medicine, Xizang Minzu University, Xianyang 712082, Shaanxi, China

**Keywords:** high altitude polycythemia, *EPHA2*, *AGT*, gene polymorphisms, case-control study

## Abstract

High altitude polycythemia (HAPC) refers to the long-term living in the plateau of the hypoxia environment is not accustomed to cause red blood cell hyperplasia. The pathological changes are mainly the various organs and tissue congestion, blood stasis and hypoxia damage. Although chronic hypoxia is the main cause of HAPC, the related molecular mechanisms remain largely unclear. This study aims to explore the genetic basis of HAPC in the Chinese Han and Tibetan populations. We enrolled 100 patients (70 Han, 30 Tibetan) with HAPC and 100 healthy control subjects (30 Han, 70 Tibetan). To explore the hereditary basis of HAPC and investigate the association between *EPHA2* with *AGT* and HAPC in Chinese Han and Tibetan populations. Using the Chi-squared test and analyses of genetic models, rs2291804, rs2291805, rs3768294, rs3754334, rs6603856, rs6669624, rs11260742, rs13375644 and rs10907223 in *EPHA2*, and rs699, rs4762 and rs5051 in *AGT* showed associations with reduced HAPC susceptibility in Han populations. Additionally, in Tibetan populations, rs2478523 in *AGT* showed an increased the risk of HAPC. Our study suggest that polymorphisms in the *EPHA2* and *AGT* correlate with susceptibility to HAPC in Chinese Han and Tibetan populations.

## INTRODUCTION

HAPC is an increased number of circulating erythrocytes develop in high altitude dwellers to compensate for high altitude associated hypoxia. The clinical diagnosis of HAPC requires a hemoglobin concentration ≥ 190 g/L for female or ≥ 210 g/L for men, and accompanied by the following three or more symptoms: dyspnea, palpitations, sleep disorders, venous dilatation, headache and tinnitus. Compared with the same altitude of healthy people, HAPC patients with red blood cells, hemoglobin and erythrocyte volume was significantly increased and oxygen saturation was decreased. Most people occur at an altitude of more than 3200 m area, but there are a few patients can occur in less than 3200 m area. The incidence of HAPC in the Tibetan Plateau is 5% to 18% [1], and the incidence of HAPC increases as the altitude increases, which is a serious public health problem in China and other Andean countries [2]. It is known that hypobaric hypoxia is the main reason for the change of pathophysiology in high altitude area, and the physiological changes of the plateau acclimatization involve oxygen intake, transportation and utilization [3]. However, there is no effective prevention or treatment measures to deal with this disease because the pathogenesis is poorly understood. The incidence of HAPC in Europe is higher than the Andean groups, and the incidence of HAPC in Andean populations is higher than the Chinese Tibetan populations who living at the same altitude. Simonson et al. [4] found the hemoglobin concentrations were significantly associated with single nucleotide polymorphisms (SNPs) of several genes in Tibetan populations, these results suggest that HAPC presents obvious racial and significant individual differences in susceptibility.

HAPC mainly leads to a significant increase in blood viscosity, causing damage to microcirculatory disturbances, vascular thrombosis, extensive organ damage, sleep disorders, and death [5]. The Qinghai-Tibet Plateau is the world's highest plateau, with an average elevation of more than 4000 meters. Most of the Tibetan people reside at altitude of 3000 m to 4500 m for a long time. Due to heritable adaptations, so they can better adapt to the hypoxia environment, as indicated by lower hemoglobin levels, lower hematocrit, higher oxygen saturation and other characteristics to help them better adapt to the plateau hypoxia environment. However, despite these genetic adaptability, the Tibetan populations still develop into HAPC in plateau area [6]. Several studies have shown that both the permanent high altitude natives and migrants show susceptibility to HAPC in the plateau, but the prevalence of HAPC among migrants was significantly higher. It is mainly due to the significant differences in the genome of the two groups, suggesting that genetic factors may contribute to the development of HAPC. Although early studies have reported the genetic basis of HAPC, only some of these genetic factors have been reported, and most studies have focused on high-altitude populations and genes involved in the hypoxia-inducible factor pathway. Growing evidence suggests that the positive directional selection of genes such as *EPAS1*, *EGLN1*, *CARD14*, *SENP1*, and *VEGFA* plays an important role in the adaptation of permanent high altitude natives and migrants [7–9]. Furthermore, we also found several genes and loci which were located chromosome 1. These genes are significantly associated with the susceptibility to HAPC, especially in the hypoxia environment of the permanent high altitude natives and migrants.

In this study, we discovered that *EPHA2*, a member of the large family of ephrin receptor tyrosine kinases, helps to maintain epidermal tissue homeostasis. *EPHA2* is overexpressed in tumors and promotes metastasis by stimulating cell migration and angiogenesis. In addition, the maintenance of genomic stability is critical to the development of organisms and the prevention of disease. Huynh-Do et al. [10] took advantage of an established model of kidney infarction to describe the effect of local renal hypoxia on *EPHA2* and erythropoietin (EPO) regulation. They found *EPHA2* increases tubular cell attachment, laminin secretion and modulates EPO expression after renal hypoxia injury. EPO is a hormone that increases the amount of red blood cells and increases the oxygen content of the blood in the human body. It contains a certain amount in the normal human body to maintain and promote normal erythrocyte metabolism. The *AGT* (rs699) genotyping of the M235T polymorphism was associated with chronic mountain sickness (CMS) in Tibetan populations living between 3600 and 4400 m, it was significantly associated with oxygen saturation in CMS patients [11]. Gao et al. [9] reported that *AGT* 235M allele showed a significant association with risk of HAPC development in Tibetans. Here, we conducted a case-control study to investigate the association of these genes variants with HAPC in Chinese Han and Tibetan populations.

## RESULTS

Table 1 shows the basic information of cases and controls groups. The basic information of candidate SNPs in Han and Tibetan subjects were summarized in Table 2 and Table 3. The location information of candidate SNPs in the case and control groups were presented in Table 4. In Han populations, we found that the rs2291804 (OR = 0.137, 95% CI = 0.035-0.536, *p* = 0.001), rs2291805 (OR = 0.293, 95% CI = 0.129-0.665, *p* = 0.002), rs3768294 (OR = 0.291, 95% CI = 0.131-0.647, *p* = 0.002), rs3754334 (OR = 0.402, 95% CI = 0.192-0.840, *p* = 0.014), rs6603856 (OR = 0.321, 95% CI = 0.140-0.737, *p* = 0.006), rs6669624 (OR = 0.359, 95% CI = 0.150-0.856, *p* = 0.017), rs11260742 (OR = 0.344, 95% CI = 0.155-0.761, *p* = 0.007), rs13375644 (OR = 0.296, 95% CI = 0.123-0.713, *p* = 0.005) and rs10907223 (OR = 0.354, 95% CI = 0.152-0.821, *p* = 0.013) in *EPHA2* were significantly associated with decreased HAPC risk. Furthermore, the rs699 (OR = 0.446, 95% CI = 0.222-0.897, *p* = 0.022), rs4762 (OR = 0.253, 95% CI = 0.091-0.701, *p* = 0.005) and rs5051 (OR = 0.413, 95% CI = 0.207-0.826, *p* = 0.011) in *AGT* gene were associated with decreased HAPC susceptibility. Similarly, in Tibetan populations, the rs2478523 (OR = 2.629, 95% CI = 1.005-6.876, *p* = 0.043) in *AGT* were associated with increased HAPC susceptibility.

**Table 1:** Basic characteristics of the control individuals and patients with high altitude polycythemia

Variables	Han		Tibetan	
	Case (n=70)	Control (n=30)	Case (n=70)	Control (n=30)
Sex				
Male	35	15	35	15
Female	35	15	35	15

**Table 2:** Basic information of candidate SNPs in Han subjects

SNP_ID	Gene	Alleles A/B	Case (N)			HWE Case	Control (N)			HWE Control	OR (95% CI)	*p* value
			AA	AB	BB		AA	AB	BB			
rs2230597	EPHA2	G/A	4	21	45	0.470	3	5	22	0.106	1.422(0.627-3.229)	0.398
rs6678616		C/T	0	4	66	1.000	1	5	24	1.000	0.312(0.081-1.206)	0.076
rs2291804		C/T	0	3	67	1.000	1	8	21	1.000	0.137(0.035-0.536)	**0.001**
rs35127370		C/T	0	13	57	1.000	1	7	22	1.000	0.746(0.281-1.976)	0.554
rs2291805		C/T	0	13	57	1.000	2	13	15	0.636	0.293(0.129-0.665)	**0.002**
rs3768294		G/A	0	14	56	1.000	2	14	14	0.390	0.291(0.131-0.647)	**0.002**
rs6678618		C/T	0	4	66	1.000	1	5	24	1.000	0.311(0.080-1.260)	0.076
rs6603883		G/A	1	17	52	1.000	1	8	21	1.000	0.981(0.403-2.388)	0.967
rs3754334		G/A	0	20	50	0.337	2	15	13	0.370	0.402(0.192-0.840)	**0.014**
rs6603856		G/A	0	13	57	1.000	2	12	16	1.000	0.321(0.140-0.737)	**0.006**
rs6669624		G/A	0	12	58	1.000	2	10	18	1.000	0.359(0.150-0.856)	**0.017**
rs11260742		T/C	1	13	56	0.568	3	11	16	1.000	0.344(0.155-0.761)	**0.007**
rs13375644		C/T	3	14	53	1.000	4	11	15	1.000	0.296(0.123-0.713)	**0.005**
rs10907223		G/A	0	13	57	1.000	2	11	17	1.000	0.354(0.152-0.821)	**0.013**
rs699	AGT	G/A	1	23	46	0.680	2	17	11	0.198	0.446(0.222-0.897)	**0.022**
rs11122576		T/C	7	35	28	0.600	3	10	17	0.644	1.692(0.845-3.389)	0.135
rs5046		G/A	3	25	42	1.000	1	8	21	1.000	1.778(0.763-4.143)	0.179
rs3789679		G/A	7	37	26	0.594	3	10	17	0.604	1.846(0.904-3.769)	0.090
rs1926722		C/A	11	31	28	0.617	3	12	15	0.662	1.469(0.759-2.844)	0.252
rs4762		G/A	1	5	64	0.146	2	8	20	1.000	0.253(0.091-0.701)	**0.005**
rs28730748		G/A	3	6	61	0.196	1	7	22	1.000	0.455(0.157-1.321)	0.140
rs2071406		A/G	1	19	50	1.000	2	8	20	1.000	0.847(0.372-1.931)	0.693
rs5050		T/G	1	13	56	0.568	2	8	20	1.000	0.576(0.242-1.37)	0.208
rs11122575		A/G	8	15	47	0.000	2	10	18	1.000	1.277(0.554-2.942)	0.565
rs3827749		G/A	3	28	39	0.744	3	14	13	1.000	0.609(0.313-1.185)	0.143
rs5051		T/C	1	23	46	0.680	2	16	12	0.413	0.413(0.207-0.826)	**0.011**
rs11568020		C/T	0	7	63	1.000	0	2	28	1.000	3.000(0.361-24.95)	0.287
rs2478523		A/G	8	28	19	0.782	5	13	12	1.000	0.841(0.431-1.638)	0.610

SNP: Single-nucleotide polymorphism; OR: odds ratio; 95% CI: 95% confidence interval; HWE: Hardy-Weinberg equilibrium;

Site with HWE *p*≤0.05 excluded; *p*<0.05 indicates statistical significance for allele model.

**Table 3:** Basic information of candidate SNPs in Tibetan subjects

SNP_ID	Gene	Alleles A/B	Case (N)			HWE Case	Control (N)			HWE Control	OR (95% CI)	*p* value
			AA	AB	BB		AA	AB	BB			
rs2230597	EPHA2	G/A	0	22	48	0.195	0	9	21	1.000	1.056(0.455-2.453)	0.898
rs6678616		C/T	0	3	67	1.000	0	2	28	1.000	0.635(0.103-3.901)	0.621
rs2291804		C/T	0	2	68	1.000	0	0	30	1.000	0.264(0.198-3.578)	0.352
rs35127370		C/T	0	14	56	1.000	0	6	24	1.000	1.000(0.365-2.74)	1.000
rs2291805		C/T	1	10	59	0.402	1	4	25	0.241	0.844(0.301-2.364)	0.746
rs3768294		G/A	3	13	54	0.480	3	4	23	0.257	0.910(0.328-2.53)	0.857
rs6678618		C/T	0	3	67	1.000	0	2	28	1.000	0.635(0.103-3.901)	0.621
rs6603883		G/A	0	18	52	0.588	0	8	22	1.000	0.959(0.392-2.344)	0.927
rs3754334		G/A	1	17	52	1.000	1	7	22	0.505	0.890(0.377-2.098)	0.790
rs6603856		G/A	2	12	56	0.519	1	5	24	0.325	0.855(0.327-2.238)	0.749
rs6669624		G/A	4	12	54	1.000	1	3	26	0.165	1.100(0.370-3.275)	0.864
rs11260742		T/C	2	11	57	0.162	1	5	24	0.325	0.910(0.350-2.356)	0.844
rs13375644		C/T	3	18	49	0.048	3	5	22	0.121	1.150(0.343-3.86)	0.821
rs10907223		G/A	1	12	57	0.514	1	5	24	0.325	0.841(0.321-2.202)	0.725
rs699	AGT	G/A	7	28	35	0.776	2	16	12	0.423	0.857(0.449-1.637)	0.640
rs11122576		T/C	5	33	32	0.573	2	14	14	0.688	1.034(0.535-1.999)	0.920
rs5046		G/A	0	13	57	1.000	0	7	23	1.000	0.775(0.293-2.051)	0.607
rs3789679		G/A	4	28	38	1.000	3	10	17	0.644	1.027(0.501-2.106)	0.942
rs1926722		C/A	5	30	35	0.777	2	14	14	0.688	0.933(0.481-1.811)	0.838
rs4762		G/A	0	6	64	1.000	0	3	27	1.000	0.851(0.206-3.52)	0.823
rs28730748		G/A	2	12	56	0.519	2	10	18	0.553	0.519(0.216-1.251)	0.139
rs2071406		A/G	2	26	42	0.720	1	9	20	1.000	1.215(0.563-2.62)	0.619
rs5050		T/G	1	7	62	0.240	0	6	24	1.000	0.618(0.210-1.822)	0.380
rs11122575		A/G	4	16	40	0.397	4	6	20	1.000	1.528(0.576-4.051)	0.392
rs3827749		G/A	1	31	38	0.093	2	10	18	0.632	1.013(0.496-2.07)	0.971
rs5051		T/C	7	30	33	1.000	2	16	12	0.423	0.917(0.481-1.746)	0.791
rs11568020		C/T	1	1	68	0.022	0	2	28	1.000	0.635(0.103-3.901)	0.621
rs2478523		A/G	6	30	34	1.000	3	12	15	0.034	2.629(1.005-6.876)	**0.043**

SNP: Single-nucleotide polymorphism; OR: odds ratio; 95% CI: 95% confidence interval; HWE: Hardy-Weinberg equilibrium;

Site with HWE *p*≤0.05 excluded; *p*<0.05 indicates statistical significance for allele model.

**Table 4:** Location information of candidate SNPs in this study

SNP_ID	Gene	Region	Position	MAF (Han)		MAF (Tibetan)	
				Case	Control	Case	Control
rs2230597		exonic	16464673	0.207	0.155	0.157	0.150
rs6678616		exonic	16475123	0.029	0.086	0.021	0.033
rs2291804		intronic	16464936	0.021	0.138	0.014	0.000
rs35127370		intronic	16474840	0.093	0.121	0.100	0.100
rs2291805		intronic	16458814	0.093	0.259	0.086	0.100
rs3768294		intronic	16456176	0.100	0.276	0.099	0.107
rs6678618	EPHA2	exonic	16475126	0.029	0.086	0.021	0.033
rs6603883		upstream	16482976	0.136	0.138	0.129	0.133
rs3754334		exonic	16451767	0.143	0.293	0.136	0.150
rs6603856		intronic	16460840	0.093	0.241	0.101	0.117
rs6669624		intronic	16460339	0.086	0.207	0.091	0.083
rs11260742		intronic	16464260	0.107	0.259	0.107	0.117
rs13375644		intronic	16461833	0.086	0.241	0.091	0.080
rs10907223		exonic	16459745	0.093	0.224	0.100	0.117
rs699		exonic	230845794	0.179	0.328	0.300	0.333
rs11122576		intronic	230846679	0.350	0.241	0.307	0.300
rs5046		upstream	230850398	0.221	0.138	0.093	0.117
rs3789679		intronic	230849694	0.358	0.232	0.246	0.241
rs1926722		intronic	230840197	0.379	0.293	0.286	0.300
rs4762		exonic	230845977	0.050	0.172	0.043	0.050
rs28730748	AGT	intronic	230845571	0.059	0.121	0.101	0.179
rs2071406		upstream	230850641	0.150	0.172	0.214	0.183
rs5050		UTR5	230849886	0.107	0.172	0.064	0.100
rs11122575		intronic	230840269	0.211	0.173	0.186	0.130
rs3827749		intronic	230841559	0.243	0.345	0.236	0.233
rs5051		UTR5	230849872	0.179	0.345	0.314	0.333
rs11568020		UTR5	230850018	0.050	0.017	0.021	0.033
rs2478523		intronic	230841509	0.400	0.442	0.330	0.158

MAF: minor allele frequency

**Figure 1:**
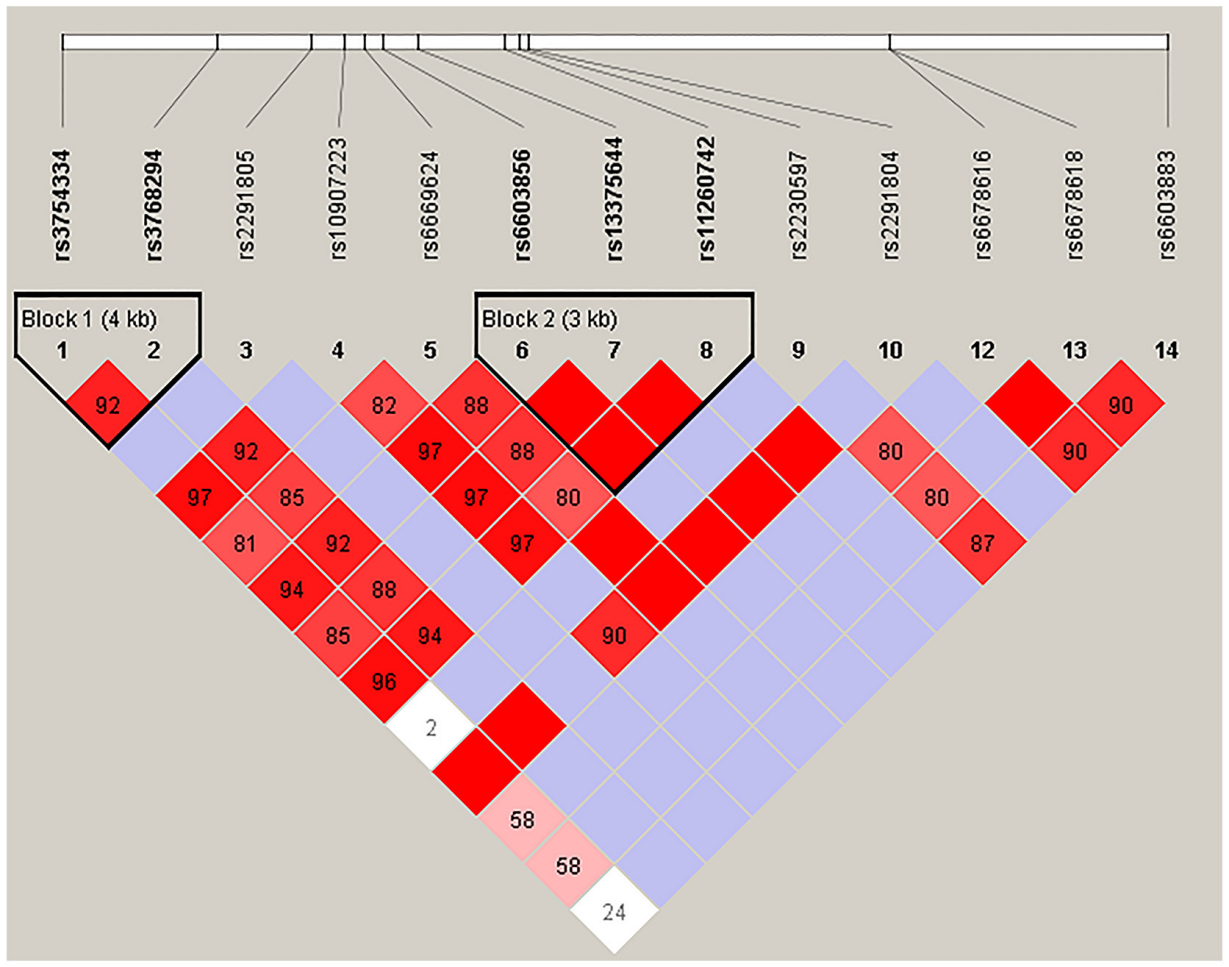
Haplotype block map for the fourteen *EPHA2* SNPs genotype in this study

**Figure 2:**
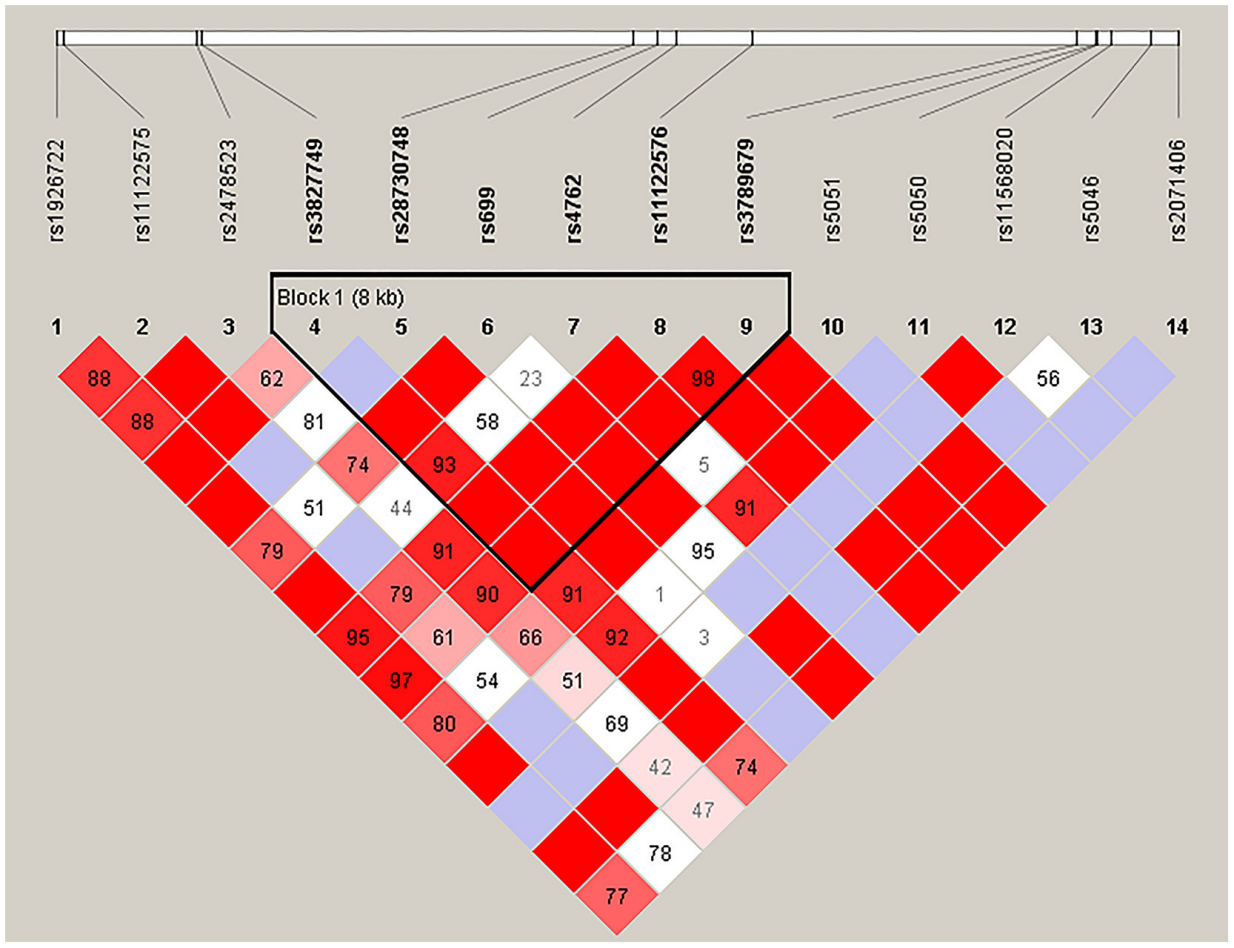
Haplotype block map for the fourteen *AGT* SNPs genotype in this study

We further analyzed the association between SNPs and HAPC risk by unconditional logistic regression analysis using three models (dominant, recessive and additive) in Han and Tibetan populations (Tables 5 and 6). After stratifying by gender, we found the rs2291805 (*p* = 0.024, *p* = 0.014), rs3754334 (*p* = 0.046, *p* = 0.025) and rs13375644 (*p* = 0.034, *p* = 0.018) in *EPHA2*, the rs699 (*p* = 0.032, *p* = 0.038), rs4762 (*p* = 0.023, *p* = 0.030) and rs5051 (*p* = 0.032, *p* = 0.021) in *AGT* were associated with a decreased risk of HAPC based on analysis using the dominant and additive model, and the rs3789679 was associated with a reduced risk of HAPC in the dominant model. Moreover, in Tibetan populations, we found the re2478523 in *AGT* (*p* = 0.016) was associated with an increased risk of HAPC in the dominant model. Furthermore, using haplotype analysis, two blocks were detected among the *EPHA2* SNPs (Figure 1). Block 1 contains rs3754334 and rs3768294, block 2 contains rs6603856, rs13375644 and rs11260742. And one block was detected among the *AGT* SNPs (Figure 2), block 1 contains rs3827749, rs28730748, rs699, rs4762, rs11122576 and rs3789679. The candidate SNPs in these genes showed strong linkages in subjects.

**Table 5:** Single loci associations with high altitude polycythemia risk in Han subjects

SNP_ID	Model	Ref Allele	Alt Allele	OR	95% CI	*p* value
rs2291805	Dominant			0.318	0.118-0.859	**0.024**
	Recessive	C	T	0.245	0.095-0.629	0.999
	Additive			0.308	0.121-0.784	**0.014**
rs3754334	Dominant			0.385	0.151-0.982	**0.046**
	Recessive	G	A	0.436	0.140-0.857	0.999
	Additive			0.361	0.148-0.878	**0.025**
rs13375644	Dominant			0.316	0.109-0.915	**0.034**
	Recessive	C	T	0.452	0.102-0.779	0.999
	Additive			0.303	0.113-0.818	**0.018**
rs699	Dominant			0.360	0.142-0.917	**0.032**
	Recessive	G	A	0.524	0.027-10.32	0.671
	Additive			0.403	0.171-0.952	**0.038**
rs3789679	Dominant			2.683	1.022-7.043	**0.045**
	Recessive	G	A	1.869	0.329-10.6	0.480
	Additive			2.088	0.956-4.561	0.065
rs4762	Dominant			0.248	0.074-0.826	**0.023**
	Recessive	G	A	0.25	0.015-4.249	0.338
	Additive			0.331	0.122-0.898	**0.030**
rs5051	Dominant			0.36	0.141-0.916	**0.032**
	Recessive	T	C	0.182	0.014-2.316	0.189
	Additive			0.377	0.165-0.862	**0.021**

OR: odds ratio; 95% CI: 95% confidence interval; *p*<0.05 indicates statistical significance for genetic model.

**Table 6:** Single loci associations with high altitude polycythemia risk in Tibetan subjects

SNP_ID	Model	Ref Allele	Alt Allele	OR	95% CI	*p* value
rs2478523	Dominant			4.533	1.325-15.500	**0.016**
	Recessive	A	G	1.095	0.200-5.988	0.917
	Additive			2.386	0.937-6.079	0.068

OR: odds ratio; 95% CI: 95% confidence interval; *p*<0.05 indicates statistical significance for genetic model.

## DISCUSSION

The Qinghai-Tibet Plateau is the largest and highest plateau in the world, which includes the largest high altitude area population. Since the Qinghai-Tibetan railway went into service, more and more Han individuals from the plain into the plateau region, the incidence of HAPC increased by 21% in Han migrants [14]. Inspired by genome research and genetic mechanism of high altitude natives, we assume that genetic factors may be involved in the pathogenesis of HAPC in Han migrants. HAPC is a serious disease in the high altitude region, especially those who have emigrated from plain area into the high altitude area. In general, the incidence of HAPC increases with the elevation of altitude. The main cause of HAPC is chronic hypoxia in the high altitude environment [15], so it is necessary to investigate the genetic basis of HAPC. Our study revealed an association between SNPs in *EPHA2*, *AGT* and HAPC in the Chinese Han and Tibetan populations. Previous studies have shown the obvious differences in the incidences of HAPC among different groups, such as Han, Tibetan and Andean populations [16]. These results suggest that HAPC has a complex pathogenesis, resulting from the interaction of environmental and genetic factors. We studied SNPs on 28 loci to be associated with an increased or decreased risk for HAPC, and were able to show statistically significant results for them.

*EPHA2* is a receptor tyrosine kinase that plays a key role in cell structure, migration and survival, upon juxtacrine contact with its membrane-bound ligand EphrinA1. In hypoxia environment, *EPHA2* is upregulated in cortical and medullary tubular cells, while EphrinA1 was upregulated in the interstitial cells adjacent to the peritubular capillaries [17, 18]. In addition, EPO messenger RNA (mRNA) was strongly expressed in the border area of the infarcted kidney within the first 6 hours. Rodriguez et al. [10] activated the signaling pathway *in vitro* using recombinant EphrinA1/Fc or *EPHA2*/Fc proteins. Stimulation of *EPHA2* positive signaling in the proximal tubular cell line HK2 increased basal-lateral cell attachment and protein secretion. In contrast, activation of reverse signalling through EphrinA1 expressed by Hep3B cells promoted EPO production at transcription and protein levels. Remarkably, intimate contact of Madin Darby Canine Kidney cells (the expression product of *EPHA2*) and Hep3B (the expression product of EphrinA1) are sufficient to induce a significant increase in EPO mRNA production in cells, even under hypoxia conditions. The synergistic effect of *EPHA2* and hypoxia results in a 15-20-fold increase in EPO expression [19]. EPO production is mainly in the kidney and liver to regulate erythrocytosis. In addition to the kidney and liver, but also the myocardium, brain and bone marrow and other organizations can detect EPO mRNA. EPO plays an important role in erythropoiesis and heart development. Moreover, EPO can prevent cardiomyocyte hypoxia-induced apoptosis. Klopsch et al. [20] showed that intracardiac injection of EPO can improve cardiac output and ejection fraction in a rat myocardial infarction model. Brunner et al. [21] reported that EPO enhanced migration of stem cells into ischemic myocardium and this was mediated through upregulation of SDF-1 expression and SDF-1/CXCR-4 pathway. Hypoxia increases the expression of EPO by HIF-1α. The most striking changes in the expression of *EPHA2* protein in acute hypoxia conditions occur in the renal medullary border, and hypoxia induced EPO expression was associated with *EPHA2* protein. EPO produced by the liver during the fetus and EPO produced by the adult kidney is identified as an inducer of erythropoiesis by stimulating erythrocytes from bone marrow differentiation precursors. EPO can promote excessive cell production, so *EPHA2* have a significant influence for the production of red blood cells.

*AGT* is an important candidate gene of the HAPC which has been shown to play a crucial role in CMS. The *AGT* gene (rs699) is located at chromosome 1 and consists of five exons, and it has more than 23 variants. The common polymorphism of the *AGT* gene is 235M, which encodes threonine instead of methionine at position 235 in exon 2. Gao et al. [22] reported *AGT* M235T (rs699) allele was associated with HAPC susceptibility in Chinese Tibetans, the specific genetic mechanism and biological functions has not been reported. However, we did not find this result in both Han and Tibetan populations. It has been also reported the *AGT* rs699 was associated with CMS in a Han Chinese population, although the description of CMS diagnosis is not clear and seems to include various disease [11]. Their results also show that the rs699 was significantly associated with the physiological parameters oxygen saturation and blood pressure among the Han Chinese populations. On the contrary, Koehle and Kalson et al. did not find this result in Nepalese and European groups, respectively [23, 24]. In addition, a significantly higher incidence of rs699 allele has been associated with atrial fibrillation in Taiwan aborigines [25]. Meanwhile, renin-angiotensin system (RAS) affects physiological and pathological effects of blood production, especially erythropoiesis. It has been reported up-regulation of local RAS, together with down-regulation of the cell surface angiotensin-converting enzyme receptors, in the autonomous neoplastic clonal erythropoiesis. The RAS was initially postulated to influence erythropoiesis following the demonstration of the haematopoietic side effects of RAS blockers [26]. Previous studies have shown the expression of angiotensin I-converting enzyme in normal erythropoietic cells and myeloproliferative bone marrow. There are several evidences suggesting the existence of local hematopoietic bone marrow RAS which contributes to the regulation of normal and disturbed hematopoiesis. So far, *AGT* has been detected in bone marrow where Ang II directly stimulates erythropoiesis through AT2R1 [27]. Based on the results of these previous studies and our research, *AGT* was significantly associated with erythropoiesis, especially in hypoxia state.

Tibet is a plateau region in Central Asia and the home to the indigenous Tibetan individuals. Tibet, an average elevation of 4900 meters, it is the highest region on earth and is commonly referred to as the “Roof of the World”. Tibetan has a unique genetic background, dietary and lifestyle habits. It has been suggested that several genetic polymorphisms are associated with susceptibility to HAPC, whereas each polymorphism may contribute to only a small relative risk of HAPC involves a complex interplay between exposure to multiple environmental stimuli and genetic background. As a unique geological condition in Central Asia, due to the difference between the area and the dietary habit, Han population has another lifestyle. This is probably the main reason for differences between Tibetan and Han populations in hereditary diseases. Although there are important discoveries revealed by the studies, there are also limitations. On the one hand, due to practical constraints, this paper cannot provide enough sample size for correlation studies. On the other hand, the functions of the genetic variants and their mechanisms have not been evaluated in this study.

## CONCLUSION

We analyzed SNPs in the *EPHA2* and *AGT* genes and identified a relationship between genetic polymorphisms and HAPC in Chinese Han and Tibetan populations. This study set out to determine paramount insights into the etiology of HAPC. However, additional genetic risk factors and functional investigations should be identified confirm our results. Finally, areas for further research are identified.

## MATERIALS AND METHODS

### Study population

After obtaining written informed consent, a total of 200 individuals from the Second People’s Hospital of Tibet Autonomous Region and Tibet military region general hospital in this study. All subjects were residing at an altitude above 4000 m for at least 3 months. According to the diagnostic criteria of CMS, we selected HAPC patients with excessive polycythemia (male, hemoglobin ≥ 210 g/L; female, hemoglobin ≥ 190 g/L) and without high altitude cerebral edema and high-altitude pulmonary edema. In addition, subjects with endocrinological, nutritional and metabolic diseases that would worsen upon hypoxemia were excluded. Healthy subjects in age and gender were randomly selected from a physical examination at an outpatient clinic to serve as controls. This research protocol was approved by the Ethics Committee of the Xizang Minzu University.

### Epidemiological and clinical data

We collected demographic and clinical data using a standardized epidemiological questionnaire, including information on age, gender, ethnicity, residential region, education status, family history of cancer. Furthermore, the case information was collected through consultation with treating physicians or from medical chart review, including blood oxygen saturation, hemoglobin and plasma erythropoietin. All participants in this study signed informed consent, and 5 ml peripheral blood was drawn from each participant.

### Selection of SNPs and methods of genotyping

Twenty-eight SNPs from two genes were chosen for analysis in this study. A total of 14 SNPs in *EPHA2* and 14 SNPs in *AGT*. Minor allele frequencies of all SNPs >5% in the Asian population HapMap database. Because the genetic basis of HAPC has not been compared between the Han ethnic groups and Tibetans, we selected candidate and SNPs based on the hypoxia inducible factor (HIF) pathway, which were associated with high altitude adaptation in the Chinese Han and Tibetan populations. Colorado et al. [12] reported *EPHA2* receptor mediates increased vascular permeability in lung injury due to viral infection and hypoxia. Furthermore, Huynh-Do et al. [10] reported the effect of local renal hypoxia on *EPHA2* and EPO regulation, they found *EPHA2* increases modulates EPO expression after renal hypoxia injury. Gao et al. [9] identified the rs699 polymorphism in the *AGT* gene was associated with HAPC susceptibility in Tibetans. Sequenom Mass ARRAY Assay Design 3.0 software was used to design multiplexed SNP Mass EXTEND assay, and SNP genotyping was performed utilizing the Sequenom Mass ARRAY RS1000 recommended by the manufacturer [13].

### Statistical analysis

Statistical analysis was performed using SPSS version 17.0 software (SPSS Inc., Chicago, IL, United States) and Excel (Microsoft Corp., Redmond, WA, United States). Hardy-Weinberg equilibrium (HWE) was calculated using SHEs is online software for patients and controls. Data are reported the proportion [odds ratio (OR)] and 95% confidence interval (CI), evaluated by three genetic models (dominant, recessive and additive) using unconditional logistic regression analysis adjusted for age and gender, and these three genetic models were performed using PLINK software and SNPStats (a web based program available at http://bioinfo.iconcologia.net/snpstats/start.htm) to assess the association of SNPs with the risk of HAPC. Multiple stepwise regression analysis was performed to assess which individual characteristic affected hemoglobin in HAPC patients, the level of entry was set at 0.05. All p values presented were calculated based on a two-sided test and statistical significance was established when p < 0.05.
